# Methylation profiling in neuropathological tumors diagnosis: a comprehensive review

**DOI:** 10.3389/fonc.2025.1720458

**Published:** 2025-12-19

**Authors:** Sarah Al Sharie, Khaled Sawaftah, Hussein Qasim, Maysa Al-Hussaini

**Affiliations:** 1 Laboratory of Science and Translation in Critical Illness, Vanderbilt University Medical Center, Nashville, TN, United States; 2 Department of Surgery, Jordan Hospital, Amman, Jordan; 3 Department of Pathology and Laboratory Medicine, Jordan University of Science and Technology, Irbid, Jordan; 4 Department of Cell Therapy and Applied Genomics, King Hussein Cancer Center, Amman, Jordan; 5 Department of Pathology and Laboratory Medicine, King Hussein Cancer Center, Amman, Jordan

**Keywords:** central nervous system neoplasms, DNA methylation profiling, ependymoma, epigenomics, glioma, medulloblastoma, meningioma, neuropathology

## Abstract

DNA methylation profiling has emerged as a transformative tool in the diagnosis and classification of central nervous system (CNS) tumors. Traditional approaches like histology, immunohistochemistry, and targeted molecular testing cannot fully capture the biological and clinical diversity of these neoplasms. In contrast, genome-wide methylation analysis provides highly reproducible epigenetic “fingerprints” that reflect both lineage and oncogenic alterations, enabling objective tumor classification, refinement of existing categories, and discovery of novel entities. This comprehensive review summarizes the principles of DNA methylation, available platforms, and the application of methylation-based classifiers across major CNS tumor groups, including diffuse gliomas, medulloblastomas, ependymomas, and meningiomas. We highlight how methylation profiling complements other molecular techniques by simultaneously generating copy number profiles and promoter methylation data, consolidating multiple diagnostic assays into a single platform. Practical considerations such as tissue quality, bioinformatic pipelines, interpretation thresholds, and cost are addressed, as are comparisons with sequencing, RNA expression, and immunohistochemistry. Finally, we explore future directions, including nanopore-based rapid testing, liquid biopsy, and artificial intelligence, which promise to extend the reach and clinical utility of methylation profiling. Collectively, these advances are establishing DNA methylation analysis as a cornerstone of precision neuropathology, aligning diagnostic and prognostic assessment with tumor biology to improve patient care.

## Introduction

The diagnosis of central nervous system (CNS) tumors has traditionally relied on microscopic evaluation of histological features, supported by immunohistochemistry and, more recently, targeted molecular assays (1). While these methods remain indispensable, it has become increasingly evident that histology alone cannot fully capture the biological diversity of CNS tumors (2). Tumors that appear identical under the microscope may differ profoundly in clinical behavior, prognosis, and therapeutic response (2). Likewise, morphologically distinct tumors may share a common molecular basis (3). Among the tools now available, DNA methylation profiling has emerged as one of the most transformative (4). DNA methylation is an epigenetic modification that regulates gene expression and reflects both the cell of origin and the genetic alterations driving tumor development (5). Each CNS tumor type, and often each subtype, carries a characteristic “methylation fingerprint” (6). These methylation signatures are highly reproducible, stable across time, and measurable on a genome-wide scale (7). The relevance of this approach extends beyond improving diagnostic accuracy (8). DNA methylation profiling has revealed new subgroups within established tumor categories, clarified the boundaries between overlapping entities, and even uncovered entirely novel tumor types (9). It has also contributed valuable prognostic insights, as certain methylation patterns correlate with clinical outcomes more strongly than histological grade alone (10). In this way, methylation analysis not only strengthens diagnosis but also enhances risk stratification and guides patient management (10). Importantly, methylation profiling does not stand in isolation but forms part of an integrated diagnostic framework (11). Rather than replacing histology, immunohistochemistry, or genetic testing, it complements them, bringing an additional dimension that ties morphological and molecular features together (12). This integrated approach improves diagnostic confidence, reduces inter-observer variability, and provides clinicians with a more reliable basis for treatment decisions (12). As the field evolves, methylation profiling is steadily moving from research into routine diagnostics (13). Its application is particularly impactful in cases where morphology is ambiguous, available tissue is limited, or standard molecular tests are inconclusive (14).

While methylation arrays can generate estimates of MGMT promoter methylation and genome-wide copy-number profiles, cIMPACT-NOW Update 9 emphasizes that these outputs must be interpreted cautiously (15). Array-derived MGMT status can be supportive of clinical decision-making but requires internal laboratory validation against established methods (e.g., pyrosequencing) due to variability in probe performance, bisulfite conversion efficiency, and algorithmic thresholds (16). Similarly, copy-number profiles derived from array intensities provide valuable contextual information—such as broad chromosomal gains/losses or focal events—but should not replace dedicated clinical-grade CNV assays unless validated locally (17).

Both MGMT methylation calls and CNV plots are particularly vulnerable to compromised sample quality (18). Low tumor cell content, extensive necrosis, or high inflammatory background can reduce probe detection rates and distort signal intensities, leading to underestimation of methylation levels or unreliable segmentation of copy-number states (19).

This review provides a comprehensive overview of the role of DNA methylation profiling in neuropathological tumor diagnosis. We will outline the principles underlying this approach, its clinical applications across major CNS tumor categories, comparisons with other molecular diagnostic methods, and the practical considerations involved in its use. Finally, we will discuss current limitations and future directions, highlighting how epigenomic profiling is shaping a new era of precision neuropathology.

## Fundamentals of DNA methylation

DNA methylation is the covalent addition of a methyl group (CH_3_) to cytosine bases, typically within CpG dinucleotides (20). Clusters of CpG sites, or CpG islands, often occur in gene promoter regions where their methylation status strongly influences transcription (21). Promoter hypermethylation is usually linked to gene silencing, while gene-body methylation has more nuanced effects (22). As a normal epigenetic mechanism, methylation regulates embryonic development, X-chromosome inactivation, and genomic imprinting (23). Each cell type carries a distinctive methylation landscape reflecting its lineage and state of differentiation (24).

In cancer, characteristic alterations arise such as the widespread hypomethylation that contributes to genomic instability, or focal promoter hypermethylation which silences tumor suppressor genes (25, 26). Importantly, tumors also retain lineage-specific methylation signatures from their cell of origin (27). The resulting composite profile, part developmental imprint, part oncogenic alteration, is highly robust, even in formalin-fixed paraffin-embedded tissue, making it a reliable diagnostic biomarker (28). For instance, IDH-mutant diffuse gliomas display the CpG island methylator phenotype (G-CIMP), while medulloblastomas, ependymomas, and meningiomas exhibit distinct methylome patterns tied to their biology (29). These tumor-specific epigenetic fingerprints often correlate more closely with behavior and prognosis than morphology alone (30).

In addition to intrinsic tumor and lineage-specific epigenetic features, the methylation profile generated by array-based platforms also reflects the contribution of non-neoplastic cells within the tumor microenvironment (31). Tumors with substantial immune infiltration, such as those enriched with macrophages, microglia, or lymphocytes, often display composite methylation signatures influenced by these cell populations, and this can shape how the classifier algorithm assigns them to a methylation class (32). These microenvironment-derived methylation patterns are particularly relevant in high-grade tumors with robust inflammatory components and underscore the importance of interpreting methylation results in the context of tumor purity and histologic background (33).

Genome-wide methylation profiling in clinical practice is most often performed using microarrays (34). Tumor DNA is bisulfite-treated, converting unmethylated cytosines to uracil while leaving methylated cytosines intact, then hybridized to platforms such as the Illumina HumanMethylation450 or EPIC 850K arrays (35). These measure methylation at hundreds of thousands of CpG sites spanning promoters, gene bodies, and regulatory elements enriched for cancer-relevant loci (36). Data are generated as quantitative β-values and analyzed bioinformatically to generate a tumor-specific methylation profile (37). Alternative methods such as targeted bisulfite sequencing or whole-genome bisulfite sequencing offer higher resolution but remain less practical for routine use (38). Arrays currently represent the best balance of coverage (~1–3% of CpGs) and cost-effectiveness in diagnostics (39). Since the introduction of the Illumina HumanMethylation450 (450K) array, methylation microarray technology has undergone two major updates aimed at expanding genomic coverage and improving assay performance. The first transition replaced the 450K with the MethylationEPIC v1 (EPIC 850K) array, which added >350,000 CpG loci enriched for enhancers and regulatory elements, while maintaining broad backward compatibility with 450K-derived datasets and classification frameworks (40). The most recent iteration, the MethylationEPIC v2 (MEP v2) array, has further refined probe chemistry, redistributed low-performing probes, and improved representation of regulatory elements, while preserving backward compatibility emphasized in cIMPACT-NOW Update 9 (41). This continuity allows laboratories to integrate new data with historical 450K and EPIC v1 datasets and ensures ongoing compatibility with widely used classifiers.

From a practical standpoint, cIMPACT-NOW 9 highlights the importance of adequate DNA input, particularly for FFPE tissue (15). While manufacturers may specify lower minimum amounts, most diagnostic laboratories recommend ≥100 ng of high-quality bisulfite-converted DNA to ensure robust signal performance, sufficient probe detection rates, and reliable copy-number output, especially in samples with variable FFPE preservation (42).

In addition to traditional bisulfite-based methods, several next-generation enzymatic methylation sequencing platforms have recently emerged. New England Biolabs’ Enzymatic Methyl-seq (EM-seq) replaces bisulfite conversion with an enzymatic oxidation/protection strategy that preserves DNA integrity, enabling more accurate and less fragmented genome-wide methylation profiling (43). Illumina’s 5-base sequencing chemistry similarly allows simultaneous detection of methylated cytosines and standard base substitutions during whole-genome sequencing, providing an integrated readout of both epigenetic and genetic alterations (44). These platforms represent an important evolution in methylation technology and are likely to become increasingly relevant as sequencing-based assays gain traction in clinical molecular pathology.

**Table 1** summarizes the major platforms currently used for DNA methylation profiling in neuropathology, highlighting their coverage, sample requirements, turnaround times, advantages, and limitations.

**Table 1:** Current DNA methylation profiling platforms in neuropathology.

Platform/method	Coverage of CpGs	Typical sample input	Turnaround time	Key advantages	Limitations/cost
Illumina 450K BeadChip	~450,000 sites (promoters, gene bodies, regulatory elements)	≥250 ng FFPE DNA	3–7 days	Historic clinical use; backbone of early classifier versions; moderate FFPE tolerance	Superseded by EPIC arrays; limited backward compatibility; reduced performance on highly fragmented FFPE DNA
Illumina EPIC 850K BeadChip (EPIC v1)	~850,000 sites (enhanced regulatory and enhancer coverage)	≥250 ng FFPE DNA	3–7 days	Expanded genome-wide coverage; robust compatibility with Heidelberg classifier (v11–v12.8)	Higher cost than 450K; some probe dropout in degraded FFPE samples
Illumina MethylationEPIC v2 (MEP v2)	~935,000 sites (updated probe chemistry; improved enhancer representation; reduced cross-reactive probes)	≥100 ng high-quality FFPE DNA (per Update 9 recommendations)	3–7 days	Improved probe performance on FFPE; higher signal-to-noise; improved CNV reconstruction; designed for backward compatibility with 450K/EPIC datasets	FFPE quality still critical; severely necrotic or low-tumor-content samples may fail QC; latest array has higher per-sample cost
Targeted Bisulfite Sequencing	Custom panels (hundreds–thousands of CpGs)	10–100 ng DNA	2–5 days	High depth at clinically relevant loci (e.g., MGMT); excellent for low-input FFPE	Limited genome-wide information; cannot support classifier-based diagnostics
Whole-Genome Bisulfite Sequencing	>95% of CpGs genome-wide	≥500 ng high-quality DNA	Multiple weeks	Comprehensive methylome; research gold standard	Not routine; resource-intensive; low feasibility for FFPE samples
Oxford Nanopore/Long-read Platforms	Genome-wide native methylation + long-range CNV	50–250 ng DNA	<48 hours	Direct methylation detection without bisulfite conversion; rapid turnaround; promising for point-of-care diagnostics	Early-stage for clinical CNS use; variable accuracy; requires local validation; performance affected by FFPE fragmentation
NEB Enzymatic Methyl-seq (EM-seq)	Genome-wide; comparable to WGBS with improved DNA preservation	50–200 ng DNA	Several days	Enzymatic conversion avoids DNA degradation; high accuracy; compatible with FFPE	Higher cost; requires high sequencing depth
Illumina 5-Base Whole-Genome Sequencing	Genome-wide methylation + base substitutions (5mC/5hmC)	≥200 ng high-quality DNA	Several days to weeks	Simultaneous methylation + mutation detection; comprehensive epigenetic/genetic profiling	Very high sequencing cost; currently limited clinical validation

## Principles of methylation-based tumor classification

The most widely used algorithm for CNS tumor methylation classification is the DKFZ/Heidelberg Molecular Neuropathology (MNP) classifier, originally developed at the German Cancer Research Center (DKFZ) and Heidelberg University. The current clinical implementation of this classifier—now commercialized through Heidelberg Epignostix—has expanded considerably beyond the initial training cohort of approximately 2,800 reference cases, incorporating many thousands of additional well-annotated tumors from both adult and pediatric populations (doi: 10.1101/2025.05.28.25328344). This expanded dataset has further refined class boundaries, improved calibration behavior, and strengthened the classifier’s ability to resolve rare or previously ambiguous entities”.

“Recent advancements have also focused on extending the versatility of methylation-based classification across platforms and biospecimen types. Notably, the MNP-Flex model (doi: 10.1038/s41591-025-03562-5) enables platform-agnostic methylation profiling, allowing reliable class assignment from data generated by different array designs and sequencing-based methylation assays. Additional parallel developments include classifier frameworks established at St. Jude Children’s Research Hospital (doi: 10.1038/s41698-024-00718-3) and the NCI Laboratory of Pathology’s Methylscape environment, both of which provide alternative structures for CNS tumor classification, benchmarking, and visualization. These complementary resources collectively broaden the diagnostic and research applicability of methylation-based tumor profiling.

Each CNS tumor type, defined by lineage and key genetic drivers, exhibits a characteristic DNA methylation signature (45). Comparing a tumor’s methylation profile to large reference datasets enables objective assignment of class or subtype (46). The seminal DKFZ classifier assembled a reference library of >2,800 brain tumors spanning 82 methylation classes, covering most recognized entities and several novel ones (6). Unsupervised clustering of these references showed that histologically defined tumors generally segregate into discrete epigenetic clusters (47). Some clusters map one-to-one to WHO entities (Category 1), others reveal molecularly distinct subgroups within single entities (Category 2, e.g., ependymoma or medulloblastoma subtypes), and still others merge previously separate pathologies or define entirely new tumor groups (48).

Operationally, the classifier uses a Random Forest model trained on these reference classes and returns calibrated scores reflecting the probability of membership in each class (49). A single top class typically emerges; scores ≥0.90 are considered confident matches (50). Intermediate scores (≈0.5–0.89) indicate uncertainty or support only a broader assignment to a *methylation class family* (MCF), for example, families encompassing multiple glioblastoma subclasses or medulloblastoma subtypes, so results can still be informative when sibling subclasses are difficult to distinguish (18, 51). Earlier versions of the classifier (e.g., v11b4 in the 2018 validation study) suggested an exploratory cutoff of 0.84; however, current practice and cIMPACT-NOW Update 9 endorse a ≥0.90 calibrated score as the general benchmark for high-confidence class assignment. Scores below ~0.5 are generally deemed unclassifiable and may reflect either truly novel biology absent from the reference set or suboptimal sample quality (13).

Importantly, cIMPACT-NOW Update 9 emphasizes that calibrated classifier scores exist on a continuum rather than representing a binary ‘match’ or ‘no-match’ outcome (15). Subthreshold scores, particularly those within a relevant methylation class family, may still meaningfully support a diagnosis when histology, immunophenotype, and molecular findings are concordant (15). Thus, classifier results should be interpreted within an integrated framework rather than rejected solely because the calibrated value falls slightly below a numerical threshold.

Different classifier implementations are currently in use, including the DKFZ/Heidelberg classifier (e.g., versions 11b4 through 12.8) and the NCI/Bethesda classifier, each with distinct training sets and class structures (52). Laboratories should specify which classifier version was applied, as updates may refine class boundaries, introduce new entities, or recalibrate probability estimates.

Current clinical practice follows classifier-specific interpretation rules. For the Heidelberg/DKFZ MNP classifier (versions 11b4 through 12.x), a calibrated score ≥0.90 is considered a high-confidence match to a specific methylation class. Scores between 0.50–0.89 support classification only at the methylation class family level, while scores <0.50 are generally considered non-classifiable. In contrast, the NCI/Bethesda Methylscape classifier uses a two-level scoring system requiring both a high super-family score and a sufficiently high class-level score for a definitive match. Specifically, the super-family score must exceed the model-defined threshold (typically ≥0.85), and the class-level score should be ≥0.90 for a high-confidence call. If the super-family score is high but the class score falls below threshold, the result should be reported as a super-family assignment only. Because reporting rules differ between classifiers, laboratories must follow the interpretation guidelines of the specific classifier used and should explicitly document classifier version, score thresholds, and match level (class, family, or super-family) in the final integrated diagnosis.

As an example:

*Histopathology:* Posterior fossa tumor with classic ependymoma morphology (perivascular pseudorosettes) and retained EMA expression.

*Methylation profiling (DKFZ/Heidelberg v12.x classifier):*

- Super-family: Posterior fossa ependymoma family calibrated score 0.94
- Best-matching class: PFA calibrated score 0.87 (below class-level match threshold; therefore reported as *family-level match only*)
- Integrated diagnosis: Posterior fossa ependymoma, PFA (family-level methylation support), WHO CNS5 criteria.

Diagnosis is based primarily on histopathologic findings and supported by methylation profiling and H3K27me3 loss.

Beyond classification, methylation arrays yield genome-wide copy number variation (CNV) profiles derived from probe intensities (53). In a single assay, one can detect hallmark alterations such as the 1p/19q codeletion in oligodendroglioma, EGFR amplification in glioblastoma, MYCN amplification, or CDKN2A/B homozygous deletions (54). This CNV layer complements the class call and adds diagnostic and prognostic value (e.g., confirming an IDH-mutant tumor with 1p/19q codeletion as oligodendroglioma) (55). Arrays can also report promoter methylation at clinically relevant loci, most notably MGMT in glioblastoma, which informs temozolomide responsiveness; although often performed by separate assays, MGMT status can be extracted from array data (56).

## cIMPACT-NOW update 9: practical recommendations for diagnostic use

cIMPACT-NOW Update 9 provides detailed guidance on when and how genome-wide DNA methylation profiling should be incorporated into clinical neuropathology (15). The update emphasizes that methylation profiling represents one diagnostic layer within the WHO/ICCR integrated reporting framework, to be interpreted alongside histology, immunophenotype, and targeted molecular testing. Reports should explicitly document the classifier version, calibrated score, and match level (“match,” “match within family,” or “no match”), thereby ensuring transparency and reproducibility across institutions (15).

The update also recommends the use of dimensionality-reduction visualization tools, such as UMAP or t-SNE, particularly for borderline or ambiguous cases (57). These plots allow the queried tumor to be visually compared to reference clusters, helping assess whether a subthreshold result is nonetheless topologically consistent with a recognized class or family (57).

Importantly, cIMPACT-NOW 9 clarifies the relationship between methylation classes and WHO tumor types, noting that some classes map directly to WHO entities while others represent biologically meaningful subgroups or families that require integration with additional data (see update 9 **Tables 1**–**3**) (15). Thus, classification should not be used in isolation but incorporated into a layered diagnostic model that clearly communicates the degree of confidence and any limitations.

**Table 2:** Characteristic methylation signatures of major CNS tumor entities.

Tumor entity (WHO CNS5)	Defining methylation features/subgroups	Correlated copy-number features (Ancillary)
Astrocytoma, IDH-mutant	G-CIMP profile; distinct from oligodendroglioma cluster	No 1p/19q codeletion; frequent ATRX loss patterns; occasional chr7 gain/chr10 loss absent
Oligodendroglioma, IDH-mutant, 1p/19q-codeleted	Highly stable oligodendroglial methylation cluster	Canonical whole-arm 1p/19q codeletion; TERTp-associated CNV patterns
Glioblastoma, IDH-wildtype	RTK I, RTK II, Mesenchymal methylation classes	+7/–10 signature; EGFR amp (RTK I); CDKN2A/B homozygous deletion
Diffuse midline glioma, H3K27-altered	Two methylation groups: DMG-K27A and DMG-K27B	PDGFRA gain (subset); focal 1q gain; absence of IDH mutation
Pediatric-type diffuse HGG, H3-WT/IDH-WT	Multiple methylation subgroups (e.g., RTK1, RTK2, MES, HPAP) – *required for diagnosis* per cIMPACT-NOW 11	PDGFRA/KIT/KDR amplifications (RTK2); broad 1q gain (HPAP)
Diffuse hemispheric glioma, H3G34-mutant	Distinct G34 methylation cluster	PDGFRA amp; chr17p loss; chr3q gain
High-grade astrocytoma with piloid features (HGAP)	Unique HGAP methylation class between PXA and HGG	CDKN2A/B deletion frequent
Medulloblastoma, WNT	Tight WNT methylation cluster	Monosomy 6; CTNNB1 mutation ancillary
Medulloblastoma, SHH	SHH methylation cluster; age-related SHH-α/β/γ/δ subgrouping	Chr9q loss (subset); MYCN/GLI2 amp
Medulloblastoma, Groups 3 & 4	Robust methylation-defined separation; multiple intermediate META-subtypes	MYC amp (Group 3); isochromosome 17q (Group 4)
Posterior fossa ependymoma, PFA	Hypermethylated PFA cluster; global H3K27me3 loss	1q gain (subset); CXorf67-negative
Posterior fossa ependymoma, PFB	Distinct PFB methylation cluster	Balanced genomes overall; fewer CNVs
Supratentorial ependymoma, ZFTA-fusion	Defined ZFTA methylation class	Focal CNVs variable
Supratentorial ependymoma, YAP1-fusion	Distinct YAP1 methylation class	Few CNVs; predominantly stable
Meningioma (methylation groups)	Four reproducible methylation risk classes: benign-1, benign-2, intermediate, malignant (not WHO entities; prognostic groups)	NF2 loss (malignant groups); CDKN2A/B loss; TERTp mutation associated

**Table 3:** Diagnostic vs prognostic utility of methylation profiling.

Tumor/subgroup	Diagnostic added value of methylation profiling	Prognostic/clinical added value
Astrocytoma, IDH-mutant	Confirms G-CIMP methylation profile; differentiates astrocytoma vs oligodendroglioma when histology/IHC are insufficient	G-CIMP–high associated with better prognosis
Oligodendroglioma, IDH-mutant, 1p/19q-codeleted	Distinct oligodendroglial methylation class confirms diagnosis when FISH/NGS results are equivocal	Stable epigenetic class with favorable outcome
Glioblastoma, IDH-WT	Distinguishes true GBM from histologically lower-grade IDH-WT tumors; differentiates RTK I/RTK II/MES methylation classes	Subclasses show outcome trends; CDKN2A/B loss, EGFRamp correlate with poor prognosis
Diffuse Midline Glioma, H3K27-altered	Defines two robust methylation subgroups (DMG-K27A/DMG-K27B), not distinguishable by histology alone	DMG-K27B associated with inferior survival
Pediatric-type Diffuse High-Grade Glioma, H3-WT/IDH-WT (cIMPACT-NOW 11)	*Diagnosis requires methylation profiling.* Separates RTK1, RTK2, MES, MYCN classes	Prognostic stratification: e.g., RTK2 often poorer outcome; MYCN class distinct biology
Diffuse Hemispheric Glioma, H3-G34 mutant	Unique methylation class confirming diagnosis when sequencing ambiguous	Distinct young-adult tumor type with intermediate outcome
High-grade Astrocytoma with Piloid Features (HGAP)	Requires methylation profiling to distinguish HGAP from anaplastic pilocytic astrocytoma or GBM	Prognosis worse than pilocytic astrocytoma
Medulloblastoma – WNT	Perfectly delineated methylation class; superior to IHC surrogate markers	Excellent prognosis, therapy de-escalation
Medulloblastoma – SHH (α/β/γ/δ)	Methylation defines biologically relevant subclusters	TP53-mutant SHH has poor prognosis
Medulloblastoma – Groups 3 & 4	Methylation profiling distinguishes these entities and their intermediate META-subtypes	Group 3: worst prognosis; Group 4: intermediate
Ependymoma – PFA	Methylation required to distinguish PFA vs PFB; identifies PFA1/PFA2	PFA universally worse prognosis vs PFB
Ependymoma – PFB	Distinct PFB methylation classes	More favorable outcomes
Ependymoma – ZFTA fusion	Methylation confirms ZFTA class, important when fusion assays inconclusive	Distinct biological behavior
Ependymoma – YAP1 fusion	Identified by methylation cluster	Generally favorable prognosis
Meningioma (methylation risk groups)	Methylation grouping outperforms WHO grading for risk prediction	Identifies high-risk and benign epigenetic classes

A practical workflow aligned with update 9 includes:

1. When to order profiling:

- When histopathological findings and/or other molecular tests (IHC, targeted sequencing, FISH) are ambiguous, discordant, or insufficient to reach a confident diagnosis.
- When the differential diagnosis includes entities where methylation profiling provides essential diagnostic or prognostic stratification (e.g., PFA vs PFB ependymoma, pediatric-type diffuse high-grade gliomas, rare methylation-defined CNS tumors).
- When conventional molecular biomarkers are negative, equivocal, or fail to explain the observed histologic features.

2. How to integrate results into layered reporting:

- Include classifier version (e.g., DKFZ/Heidelberg v12.8, NCI/Bethesda), calibrated score, match level;
- Add visualization output (UMAP/t-SNE) when results are borderline;
- Clearly state whether the methylation class corresponds to a WHO entity or to a broader class family.

3. Management of low-confidence or discordant results:

1. Reassess technical quality and tumor purity.

Low tumor content, FFPE degradation, necrosis, or poor probe detection can all produce subthreshold classifier scores. Re-examining the selected tissue block and repeating extraction or macrodissection is often necessary.

2. Evaluate biological concordance with histology, immunophenotype, and known driver alterations.

This step is of primary importance. A methylation result—especially a low-confidence or borderline one—must be interpreted in light of the tumor’s morphology, IHC profile (e.g., IDH1 R132H, H3K27me3, SMARCB1), and molecular findings (e.g., IDH mutation, 1p/19q codeletion, ZFTA/YAP1 fusion). Concordance or discordance here strongly determines the validity of the classifier output.

3. Manually review the CNV profile for consistency with the suspected entity and other diagnostic data.

CNV plots derived from methylation arrays must be interpreted manually, as no automated scoring or decision system exists. The goal is to assess whether observed chromosomal alterations (e.g., 1p/19q codeletion, +7/–10, CDKN2A/B homozygous deletion, MYCN amplification) support or contradict the histology and methylation class family. This is a correlation exercise—not an automated validation step.

4. If discordance persists, report the case transparently as low-confidence or uncertain.

Following WHO/ICCR layered reporting and cIMPACT-NOW 9, such cases should be documented with explicit mention of which diagnostic layers are supportive, conflicting, or inconclusive.

## Clinical applications in neuropathology

### Diffuse gliomas

Diffuse gliomas comprise adult-type astrocytomas and oligodendrogliomas, as well as pediatric-type diffuse gliomas (58). WHO 2021 stratifies them by key alterations such as IDH mutation, 1p/19q codeletion, and histone mutations (1). Methylation profiling provides strong support for this framework (59). Adult-type gliomas segregate into three entities: astrocytoma, IDH-mutant; oligodendroglioma, IDH-mutant and 1p/19q-codeleted; and glioblastoma, IDH-wildtype (60). The classifier distinguishes these with high fidelity, since IDH mutations induce a CpG island methylator phenotype absent in IDH-wildtype glioblastomas (61). Oligodendrogliomas form a separate cluster, reinforced by detection of the 1p/19q codeletion on copy number plots (62). IDH-mutant astrocytomas often display a “G-CIMP-high” profile, correlating with favorable prognosis (63). **Table 2** summarizes the characteristic methylation signatures and ancillary genetic hallmarks of major CNS tumor entities currently defined by the 2021 WHO classification.

In glioblastomas, methylation has revealed biologically distinct subgroups such as RTK I, RTK II, and Mesenchymal, which parallel transcriptional subtypes (64). While not yet guiding therapy, these have prognostic trends and may influence future management (65). Methylation profiling is particularly useful for identifying IDH-wildtype tumors that histologically resemble lower-grade astrocytomas but biologically align with glioblastoma. According to WHO CNS5, if methylation profiling and integrated diagnostic data support a glioblastoma-type methylation class or demonstrate characteristic glioblastoma-associated molecular alterations, the tumor should be designated as ‘Glioblastoma, IDH-wildtype, WHO grade 4,’ rather than ‘Diffuse astrocytic glioma, IDH-wildtype, with molecular features of glioblastoma.’ The latter terminology is obsolete and no longer used in contemporary practice (66). The classifier can also act as a surrogate when genetic testing is unavailable, though confirmation of critical mutations remains advisable (66).

In pediatric gliomas, methylation profiling defined entities such as diffuse midline glioma, H3 K27-altered, and diffuse hemispheric glioma, H3 G34-mutant (67). It also contributed to delineating the broader group of pediatric high-grade gliomas, which contain multiple methylation-based subsets under study (68). Even in low-grade pediatric gliomas, methylation aids in clarifying diagnosis (69). For example, distinguishing *BRAF* fusion positive diffuse low-grade glioma from more aggressive infant-type hemispheric gliomas with RTK fusions (70, 71).

Recent methylation analyses have shown that diffuse midline gliomas, H3K27-altered, segregate into at least two robust methylation subgroups (DMG-K27A and DMG-K27B), with distinct developmental and clinical implications (72). These refinements are not captured by histopathology or single-gene assays, emphasizing the essential role of methylation profiling for accurate subclassification.

### Medulloblastomas

The application of DNA methylation-based classification to medulloblastoma exemplifies how epigenomic profiling can profoundly reshape tumor taxonomy (73). WHO now recognizes four core molecular subgroups: WNT-activated, SHH-activated (TP53-wildtype or mutant), Group 3, and Group 4 (74). Methylation profiling robustly assigns tumors to these subgroups and consistently outperforms immunohistochemical surrogates (75).

WNT-activated tumors, which frequently harbor CTNNB1 mutations and monosomy 6, form a tight epigenetic cluster and have an excellent prognosis, supporting treatment de-escalation strategies (76). SHH-activated tumors, driven by pathway alterations and further subdivided by TP53 status, demonstrate variable outcomes; TP53-mutant pediatric SHH tumors in particular carry a poor prognosis (77). Within the SHH group, additional methylation-defined subclusters (α–δ) capture age-related and genetic differences, although these remain investigational (78).

Group 3 and Group 4 tumors—historically merged under a single category—were first differentiated by methylation profiling, which revealed distinct biological and clinical behaviors (79). Group 3 tumors, often characterized by MYC amplification, have the worst prognosis, whereas Group 4 tumors, frequently associated with features such as isochromosome 17q, are more common and exhibit intermediate outcomes (80).

It is important to note that the foundational subdivision of medulloblastoma into WNT, SHH, Group 3, and Group 4 was initially established through integrative transcriptomic and copy-number analyses, particularly via gene-expression array profiling (81). Subsequent DNA methylation-based classification reproduced these biologically meaningful subgroups with high fidelity and provided a more stable, clinically scalable framework for diagnostic subgroup assignment.

### Ependymomas

DNA methylation profiling was central to establishing the modern molecular taxonomy of ependymomas, enabling the separation of posterior fossa tumors into the biologically distinct PFA and PFB groups and redefining several supratentorial categories (82). In the supratentorial compartment, methylation distinguished *ZFTA* fusion–positive tumors from *YAP1* fusion–positive ones, which differ in biology and prognosis (83). In the posterior fossa, methylation studies identified two major groups: PFA, hypermethylated and aggressive with H3K27me3 loss, and PFB, associated with older patients and better outcomes (84). These cannot be separated reliably by histology, making DNA methylation the diagnostic gold standard (84).

It is important to note that the original separation of posterior fossa ependymomas into the PFA and PFB subgroups was first identified through transcriptome-based stratification using expression array profiling (85). Subsequent DNA methylation profiling reproduced these two core groups with high concordance and provided a more robust, reproducible, and clinically applicable framework for classification (85). This alignment between expression-based and DNA methylation–based clustering helped establish PFA and PFB as biologically and clinically distinct disease entities.

Spinal ependymomas usually show *NF2* alterations, but methylation identified a distinct, aggressive *MYCN*-amplified subtype, now a separate WHO entity (86). Importantly, methylation arrays simultaneously detect fusion status, CNVs, and subgroup assignment, often resolving diagnostic ambiguities (87).

DNA methylation profiling is essential for accurately distinguishing PFA from PFB posterior fossa ependymomas, a separation that cannot be reliably achieved by histology, immunohistochemistry, or targeted sequencing alone (88). PFA tumors show a characteristic hypermethylated profile and loss of H3K27me3, whereas PFB tumors cluster separately and maintain H3K27me3 expression (89). However, classifier accuracy is highly dependent on tumor purity: low-cellularity samples, biopsies with abundant reactive tissue, or tumors with extensive necrosis frequently produce subthreshold scores or ambiguous class-family assignments (90). Furthermore, cIMPACT-NOW Update 11 provides updated diagnostic criteria for both posterior fossa ependymoma and pediatric-type diffuse high-grade gliomas (IDH-wt, H3-wt), emphasizing the importance of integrated molecular–methylation analysis for entities previously categorized as “NOS” or “NEC” (91).

### Meningiomas

Histologic grading has limited predictive power for meningiomas, as some grade 1 tumors recur aggressively while others remain indolent (92). Methylation studies have proposed biologically driven risk groups, now confirmed across multiple cohorts (93). Classes include benign Merlin-intact tumors with low recurrence risk, immune-enriched tumors with intermediate behavior, and proliferative or hypermetabolic tumors with poor prognosis (94). These classes align partly with genetic features such as *NF2*, *CDKN2A/B*, and *TERT* promoter mutations (95).

Methylation-based stratification predicts recurrence more accurately than histologic grade alone, especially within grade 2 meningiomas, where outcomes vary widely (96). This has major clinical implications for postoperative management, influencing decisions regarding surveillance or adjuvant therapy (97). While not yet formalized in WHO 2021, methylation groups are likely to be incorporated into future classification systems, given their superior prognostic performance and therapeutic relevance (15).

Recent studies further demonstrate that methylation-based risk groups consistently outperform conventional WHO grading in predicting recurrence, even when adjusting for extent of resection (96). These risk groups should be interpreted in conjunction with key molecular alterations, most notably CDKN2A/B homozygous deletion and TERT promoter mutations, both of which designate WHO grade 3 irrespective of histology and strongly correlate with high-risk methylation classes (98).

In addition, cIMPACT-NOW Update 8 reinforces the central role of DNA methylation profiling in meningioma classification and recommends its integration into routine diagnostic practice, particularly for tumors with borderline histologic features or discordant clinical behavior (99).

### Rare and ambiguous CNS tumors

Methylation profiling is particularly powerful in rare or diagnostically uncertain tumors (59). Historically vague categories, such as CNS primitive neuro-ectodermal tumors, have been redefined through methylome analysis into distinct entities like CNS neuroblastoma, FOXR2-activated, and CNS tumor with BCOR internal tandem duplication (100). Similarly, astroblastoma, *MN1*-altered, and several pediatric glioneuronal tumors (e.g., PLNTY, DGONC) were delineated through methylation-based clustering (101).

The approach also refined existing categories, as with atypical teratoid/rhabdoid tumors (AT/RT), where methylation identified TYR, SHH, and MYC subgroups with different clinical associations (102). For diagnostically ambiguous or “NOS” cases, methylation often provides a definitive match, guiding clinical management (103). Even when no exact class match is achieved, methylation data can place tumors within a broader family, offering useful diagnostic direction (104). Importantly, many new CNS tumor types recognized in WHO 2021 originated from such “no match” clusters in methylation space, underscoring the method’s role in discovery as well as diagnosis (6).

Collectively, these examples demonstrate how methylation profiling not only clarifies histologic diagnosis but also provides prognostic and sometimes predictive information across tumor types. **Table 3** summarizes the diagnostic and prognostic contributions of methylation profiling across key CNS tumor entities.

## Practical considerations in the diagnostic workflow

**Figure 1:**
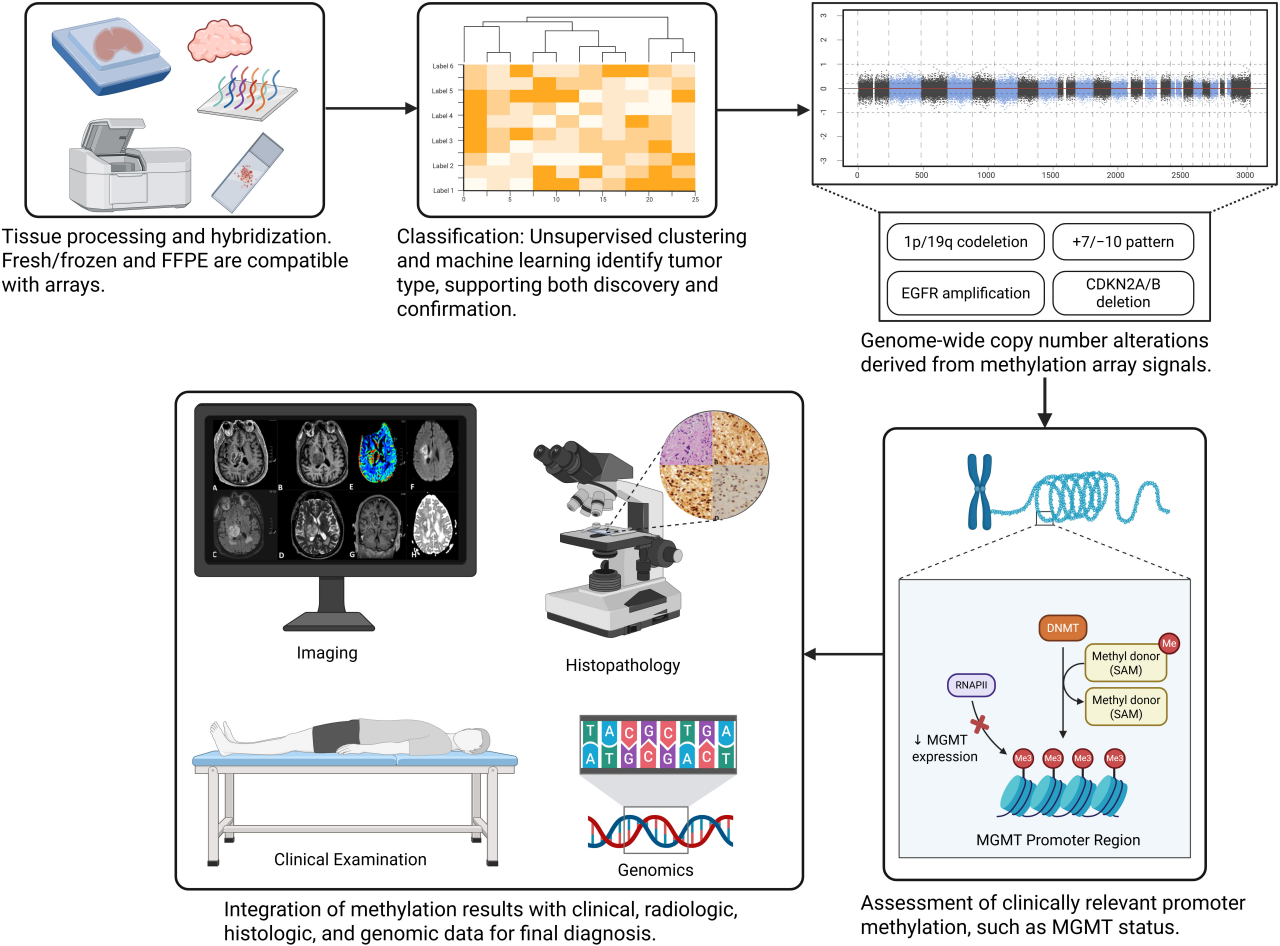
Schematic overview of how DNA methylation profiling is implemented in clinical neuropathology. DNMT, DNA methyltransferase; SAM, S-adenosyl-methionine; MGMT, O-6-methylguanine-DNA methyltransferase; RNA Pol II, RNA polymerase II; Me, methyl group.

Implementing DNA methylation profiling in diagnostic neuropathology requires attention to technical, logistical, and interpretive aspects (4). Adequate tissue and DNA quality are fundamental (4). While methylation arrays can be performed on Formalin-Fixed Paraffin-Embedded (FFPE) tissue, the DNA must not be too degraded and should be available in sufficient quantity, typically >250 ng for the Illumina EPIC 850K array (105). Low tumor content poses difficulties, as background signal from non-neoplastic brain or inflammatory cells can obscure the tumor profile (106). Small biopsies or diffuse gliomas with sparse cellularity are especially problematic (68). Macrodissection of slides to enrich tumor tissue is therefore recommended before DNA extraction (107). **Figure 1** provides a simplified overview of where DNA methylation profiling fits within the broader diagnostic workflow, illustrating how histopathology, immunohistochemistry, targeted sequencing, and methylation-based classification are integrated into routine practice.

The standard workflow includes DNA extraction, bisulfite conversion, amplification and hybridization to the array, scanning and data processing, and classification (108). In practice, this takes 3–7 days, with final reports available in 1–2 weeks (108). Some laboratories batch samples for efficiency, which may introduce minor delays (109). As a result, methylation profiling generally informs the finalized diagnostic report and postoperative planning, rather than intraoperative decisions (110). Efforts are underway to shorten turnaround times using nanopore sequencing, which can generate methylation data within hours or days, though this is not yet routine in clinical laboratories (111).

Cost and availability remain important considerations (112). A single array test may cost several hundred to over one thousand US dollars, and not all hospitals have access to on-site facilities (113). Consequently, many samples are referred to specialized centers (113).

Each array includes probes that monitor bisulfite conversion efficiency and hybridization quality, and laboratories must ensure these falls within acceptable limits (114). Batch effects are another challenge, as running samples at different times or on different array models can introduce minor variations (115). Although the classifier is designed to accommodate these, best practice is to run samples in consistent batches with controls (116). Bioinformatics support may still be needed for data handling and quality control, but the Heidelberg and Methylscape classifiers automatically generate key outputs, including copy-number plots, so manual CNV computation is generally not required (117). Many centers have automated pipelines to standardize analysis and reduce variability (118).

Interpretation of classifier results is essential for determining whether the DNA methylation profile reliably supports a specific CNS tumor class and for integrating this information with morphology, immunohistochemistry, and molecular findings (119, 120). The output from the DKFZ/Heidelberg Molecular Neuropathology (MNP) classifier, currently the most widely used diagnostic tool, is provided as a set of calibrated scores representing the confidence of assignment to each reference methylation class (18). These calibrated scores form a continuum of classifier confidence rather than a strict binary decision, and their interpretation requires attention to class-specific thresholds, match levels, and ancillary data (18). Because subsequent steps in the diagnostic workflow (including reporting of match level and the use of score cutoffs) depend directly on this classifier behavior, clear understanding of the classifier’s output structure is critical (14).

A score ≥0.90 is typically considered a confident assignment, while scores between 0.5 and 0.89 suggest a possible but less certain match, often only at the level of a broader methylation class family (120). Scores below 0.5 are usually regarded as unclassifiable, possibly indicating novel biology or poor sample quality (18). Importantly, the relative distribution of scores matters: a top score of 0.93 with all others near zero provides strong confidence, whereas similar scores across multiple classes indicate uncertainty or a tumor with intermediate features (121).

Despite its robustness, classifier output must always be interpreted within clinical and histopathologic context (122). Even a high score can occasionally be misleading, especially in cases with heavy inflammatory infiltration or unusual morphology (123). The cIMPACT-NOW consortium emphasizes integration, recommending that methylation findings be reported alongside histology, immunohistochemistry, and molecular data (15).

Finally, the integration of methylation profiling requires a multidisciplinary approach. Molecular tumor boards increasingly review methylation findings in conjunction with genomic and histopathologic data (124). Pathologists often present classifier scores, copy number profiles, and clustering results to explain diagnostic changes to oncologists and neurosurgeons (14). This collaborative discussion builds confidence in the method and facilitates clinical translation (14). As experience grows, methylation profiling is transitioning from a research tool to an essential diagnostic pillar, particularly for difficult cases and novel entities (14).

## Comparisons with other molecular diagnostic approaches

DNA methylation profiling is a powerful diagnostic tool that complements rather than replaces other molecular techniques (59). Sequencing remains essential for detecting specific mutations such as *IDH1/2*, *TERT* promoter, *BRAF* V600E or rare fusions, which drive therapy choices and clinical trial eligibility (125). Methylation arrays do not directly identify sequence changes but reveal the downstream epigenetic effects of genetic events and can reclassify tumors when conventional tests give ambiguous or conflicting results (126). For example, IDH1 immunostaining and 1p/19q FISH may suffice for classic oligodendroglioma, but methylation profiling helps resolve contradictory findings or uncover unexpected entities such as ependymoma profiles in presumed glioblastoma (127).

Compared with RNA expression profiling, methylation analysis is more stable and reproducible, especially in FFPE tissue, and provides additional copy number information (128). While expression data can still be useful for pathway analysis, methylation has largely replaced expression arrays for CNS tumor classification, with integrated approaches that combine methylation, mutation, and expression now under study (6).

Methylation arrays also yield genome-wide copy number plots with high concordance to traditional cytogenetic techniques, reliably detecting hallmark alterations such as 1p/19q codeletion, +7/–10, *EGFR* amplification, or CDKN2A/B deletion (129). This often reduces the need for separate FISH or chromosomal microarray testing, although focal or cryptic alterations may still require targeted assays (130). Immunohistochemistry, meanwhile, remains indispensable for rapid, inexpensive detection of protein-level changes like IDH1 p.R132H, H3K27M, or SMARCB1 loss, but methylation offers more objective classification and deeper subclassification when staining is equivocal (131).

Emerging single-cell and spatial methods promise unprecedented resolution of intratumoral heterogeneity and epigenetic diversity but are not yet part of routine diagnostics (132). Currently, methylation profiling serves as a practical “broad brush” tool, consolidating classification, copy number analysis, and some immunohistochemical surrogates in a single assay, while sequencing adds precise mutational data (133). Together these technologies are moving the field toward integrated molecular neuropathology reports that unite genotype and epigenotype to guide diagnosis, prognosis, and treatment planning (129, 130, 132). **Table 4** compares methylation profiling with sequencing panels, RNA expression profiling, and immunohistochemistry, emphasizing their respective strengths and limitations in clinical neuropathology.

**Table 4:** Comparison of methylation profiling with other molecular approaches.

Feature	Methylation profiling	Sequencing panels	RNA expression	Immunohistochemistry
Primary Data	Epigenetic signature (CpG β-values) + CNV	DNA mutations/fusions	Gene expression levels	Protein expression
Sample Stability	High in FFPE	High	Moderate (RNA degradation)	High
Scope	Genome-wide classification + CNV + promoter methylation	Targeted mutations/fusions	Pathway activity/subtypes	Single protein targets
Turnaround Time	1–2 weeks (arrays)	5–10 days	Variable	Hours–days
Key Strength	Objective tumor class assignment	Direct mutational status for therapy	Pathway insights	Rapid, inexpensive screening
Key Limitation	Cannot call mutations directly	Limited to panel content	Less reproducible in FFPE	Surrogates may be equivocal

## Practical summary of best-practice recommendations for diagnostic methylation profiling

1. Ensure adequate tissue and DNA quality.

Optimal performance of methylation arrays requires sufficient material and high-quality DNA (134). For EPIC v1 and v2 platforms, at least 100 ng of well-preserved FFPE DNA is generally recommended, as suboptimal or heavily degraded DNA is associated with low-confidence classifier outputs (41). Tissue regions used for extraction should contain a high proportion of viable tumor cells, ideally 70% or greater, because excessive non-neoplastic tissue, treatment effect, or necrosis reduces the tumor signal and undermines both calibrated scores and CNV profiles (135).

2. Use macrodissection to enhance tumor enrichment.

Guided macrodissection of H&E-marked slides remains a simple but essential step for ensuring tumor purity (107). Removing admixed normal brain, vascular tissue, calcifications, hemorrhage, or necrotic areas helps maintain epigenetic signal quality and prevents dilution of the tumor-specific profile (136). In infiltrative gliomas, selecting regions with cytologic atypia or increased proliferation improves classifier performance and minimizes the risk of underrepresentation of the neoplastic component (136).

3. Apply rigorous pre-analytical quality control.

Pre-array quality control measures, including ΔCt thresholding, assessment of bisulfite conversion efficiency, and evaluation of DNA integrity, are crucial for determining whether a sample is suitable for reliable methylation analysis (134). Poor-quality DNA frequently leads to subthreshold calibrated scores or uninterpretable profiles (137). When QC metrics fall below acceptable levels, repeating the extraction or selecting an alternative block is preferable to proceeding with technically inadequate material (138).

4. Select cases thoughtfully to maximize clinical utility.

Methylation profiling should be applied selectively in cases where it is expected to change diagnostic interpretation (139). It is most valuable in scenarios where morphology and immunohistochemistry are ambiguous or conflicting, where essential molecular biomarkers yield negative or indeterminate results, or where the differential diagnosis includes pediatric-type high-grade gliomas, posterior fossa ependymoma subgroups, or rare and emerging methylation-defined entities such as HGAP, HPAP, or BCOR-altered tumors (89). Conversely, when a diagnosis is straightforward based on morphology, immunophenotype, and targeted sequencing, or when the biopsy is too small or dominated by necrosis, methylation profiling offers limited benefit and may not be appropriate (140).

5. Interpret results within a layered and integrated diagnostic framework.

Methylation classifier outputs should be interpreted within the WHO/ICCR layered reporting structure and understood as a continuum rather than a binary outcome (46). Each report should specify the classifier version used (for example, Heidelberg v12.8 or NCI/Bethesda), the calibrated score, and the level of match achieved, whether “match,” “family match,” or “no-match” (15). In borderline cases or subthreshold matches, dimensionality-reduction tools such as UMAP or t-SNE can help visualize relationships to reference classes (141). Ultimately, the methylation result must be reconciled with morphology, immunohistochemistry, targeted sequencing, and tumor location to achieve a coherent integrated diagnosis (142).

6. Maintain expert neuropathologic oversight.

Despite the increasing diagnostic power of methylation profiling, expert pathology review remains indispensable (143). Array results cannot substitute for comprehensive assessment of histology and ancillary molecular data (143). In cases where the classifier output conflicts with morphologic or genetic findings, the final interpretation should prioritize integrated reasoning rather than a single modality (144). The role of the neuropathologist is therefore central to ensuring that methylation data complements, rather than replaces, traditional diagnostic expertise. As summary, **Table 5** presents common pitfalls in methylation profiling and troubleshooting approaches.

**Table 5:** Common pitfalls in methylation profiling and troubleshooting approaches.

Pitfall	Impact on result	How to recognize It	Recommended troubleshooting
Low Tumor Purity	Low calibrated scores, “no-match,” distorted CNV profile	Failure of expected markers; widespread flat CNV	Repeat macrodissection; use a block with higher tumor content; consider complementary NGS
Poor DNA Quality/Degradation	Failed bisulfite conversion; high ΔCt; array failure	QC warnings; high failure probes	Re-extract DNA; choose alternative block; avoid heavily necrotic tissue
Batch Effects	Shifts in UMAP/t-SNE position leading to false “family match”	Sample clusters away from expected class	Ensure uniform processing; include controls; interpret with pathologic features
Classifier Version Mismatch	Inconsistent results compared to prior cases	Reported version differs (e.g., v12.5 vs v12.8)	Always document classifier version; re-run with most current release
Overreliance on Single Modalities	Misclassification, especially in borderline scores	Discordance with histology/IHC	Integrate morphology + IHC + NGS; treat classifier as one diagnostic layer
Small Biopsies/Limited Tissue	Non-diagnostic output; low DNA	Often high background noise	Reserve methylation profiling for cases where sufficient tissue is available
Unusual Entities Not in Classifier	“No-match” or ambiguous placement	Low or intermediate calibrated score	Consult emerging-entity literature; use WHO/ICCR layered reporting

**Figure 2:**
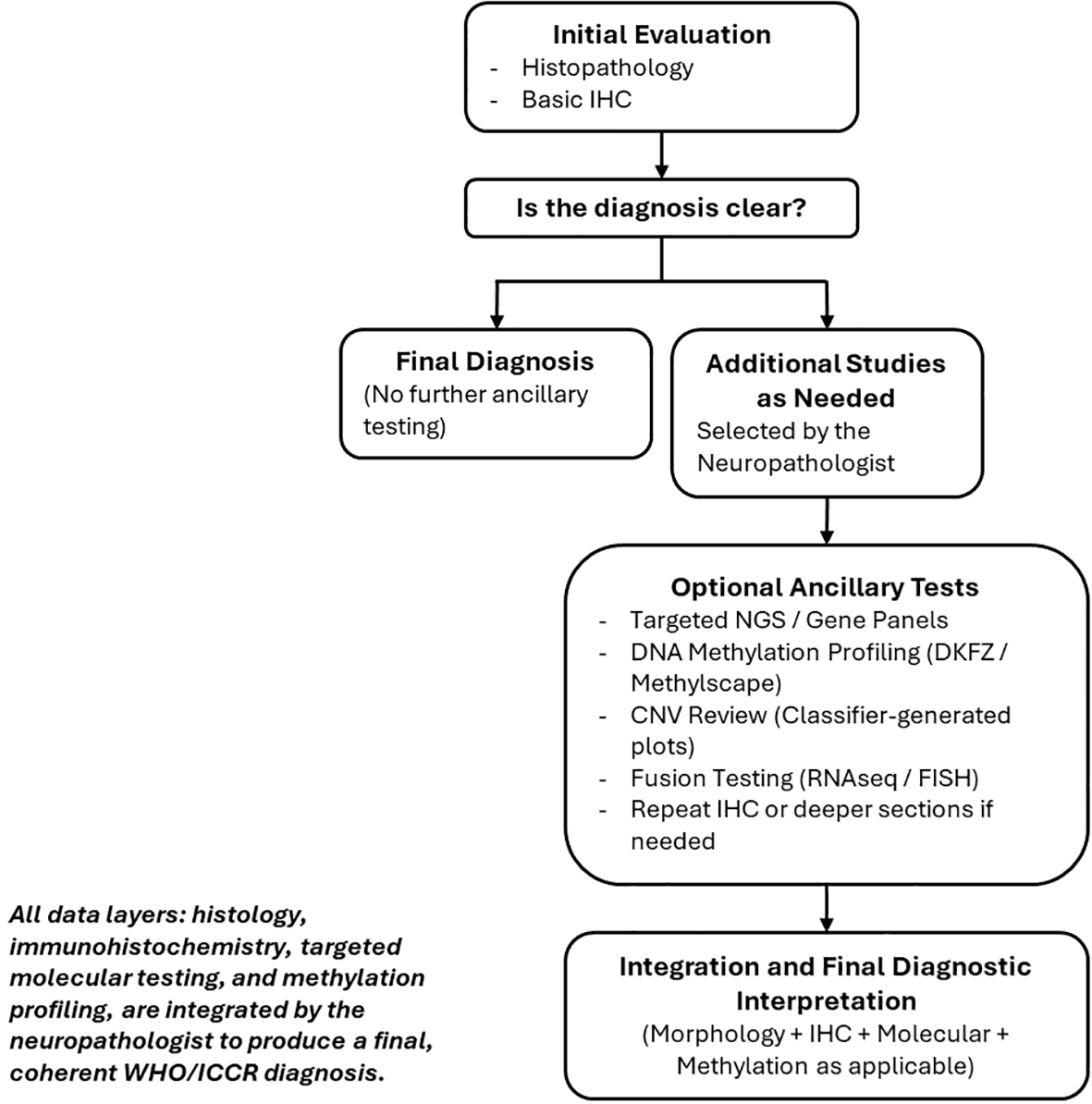
Practical diagnostic workflow illustrating how histopathology, immunohistochemistry, targeted molecular testing, and DNA methylation profiling are combined in routine neuropathology practice.

The central role of the neuropathologist in synthesizing histologic, immunophenotypic, molecular, and methylation data is outlined in **Figure 2**.

## Future directions

The field of methylation profiling in neuropathology is advancing rapidly, promising greater diagnostic precision and broader clinical use (145). As more CNS tumors, including rare and underrepresented types, are profiled worldwide, reference libraries will expand, enabling classifiers to add new tumor categories and refine existing groups into more homogeneous subtypes (146). Larger datasets will also improve discrimination between closely related entities, provided that new array- or sequencing-based platforms maintain backward compatibility with existing data (147).

Emerging sequencing-based methylation technologies are likely to broaden the diagnostic utility of methylation profiling in the near future. Enzymatic Methyl-seq (EM-seq), by avoiding bisulfite-induced DNA damage, offers higher-quality data from FFPE samples and may become a preferred approach for genome-wide methylation profiling (148). As these technologies mature and undergo clinical validation, they may provide a unified workflow for comprehensive molecular diagnostics.

While methylation arrays remain the current standard, sequencing approaches are gaining momentum (149). Whole-genome bisulfite sequencing offers comprehensive coverage but is resource-intensive, whereas reduced-representation or targeted bisulfite sequencing may provide efficient coverage of key CpGs (150). Long-read platforms such as Oxford Nanopore can directly detect methylation without bisulfite conversion, potentially integrating methylation class, copy number, and mutational data within 24–48 hours (151). With falling costs and more mature bioinformatics pipelines, sequencing-based methylation may supplant arrays over the next decade (152).

Liquid biopsy is another frontier (153). Because each tumor class exhibits a distinctive methylation signature, sensitive assays applied to plasma or cerebrospinal fluid could allow diagnosis without surgical biopsy and enable non-invasive monitoring (154). Early studies in gliomas and medulloblastomas show that CSF-derived DNA can be classified successfully, particularly valuable for high-risk lesions such as diffuse midline gliomas, although detecting low-abundance tumor DNA remains challenging (155).

Moreover, Deep-learning models trained on routine H&E slides already predict some mutations and could be extended to infer methylation classes directly from morphology, offering rapid triage or screening (156). Beyond reproducing current classifiers, AI may identify novel prognostic methylation features and integrate them into digital pathology workflows to enhance precision (157).

Finally, methylation-defined subgroups open opportunities for therapeutic stratification (158). Epigenetically defined glioblastoma or medulloblastoma subtypes may respond differently to targeted or immune therapies, just as MGMT promoter methylation predicts temozolomide response (159). The success of CNS classifiers is spurring development in other tumor systems, including pediatric sarcomas, renal tumors, and nerve-sheath tumors, with the longer-term goal of pan-cancer methylation classifiers that can pinpoint tissue of origin or distinguish primary CNS tumors from metastases (6).

## Conclusions

DNA methylation profiling has transformed neuropathological diagnosis by providing an objective and reproducible method for classifying CNS tumors. Unique epigenetic signatures now define more than a hundred tumor types; many incorporated into WHO classifications. This approach improves diagnostic accuracy, clarifies ambiguous cases, and enhances prognostic stratification, with clear benefits shown in gliomas, medulloblastomas, ependymomas, and meningiomas. Its greatest strength lies in an integrated framework, where methylation complements histology, immunohistochemistry, and sequencing. Beyond classification, arrays deliver valuable ancillary data such as copy number and promoter methylation, consolidating multiple analyses into one. Methylation profiling occupies a distinct niche: it does not replace mutation testing or rapid immunohistochemistry, but it provides a genome-wide epigenetic context that unifies diverse findings into a coherent diagnosis. Looking forward, advances in sequencing, liquid biopsy, and AI will expand its speed, accessibility, and clinical relevance. Ultimately, methylation profiling is establishing itself as a core pillar of precision neuropathology, aligning diagnosis and prognosis with tumor biology to improve patient care.
